# Characteristics of *SlCML39*, a Tomato Calmodulin-like Gene, and Its Negative Role in High Temperature Tolerance of *Arabidopsis thaliana* during Germination and Seedling Growth

**DOI:** 10.3390/ijms222111479

**Published:** 2021-10-25

**Authors:** Haidong Ding, Ying Qian, Yifang Fang, Yurong Ji, Jiarong Sheng, Cailin Ge

**Affiliations:** 1 Joint International Research Laboratory of Agriculture and Agri-Product Safety of Ministry of Education of China, Yangzhou University, Yangzhou 225009, China; 2 College of Bioscience and Biotechnology, Yangzhou University, Yangzhou 225009, China; 18852721947@163.com (Y.Q.); fyf010102@163.com (Y.F.); jyr0806@163.com (Y.J.); myyjjfb@163.com (J.S.)

**Keywords:** *Arabidopsis thaliana*, calmodulin-like protein, high temperature, RNA sequencing, *SlCML39*, tomato

## Abstract

Calmodulin-like (CML) proteins are primary calcium sensors and function in plant growth and response to stress stimuli. However, so far, the function of plant CML proteins, including tomato, is still unclear. Previously, it was found that a tomato (*Solanum lycopersicum*) CML, here named *SlCML39*, was significantly induced by high temperature (HT) at transcription level, but its biological function is scarce. In this study, the characteristics of *SlCML39* and its role in HT tolerance were studied. *SlCML39* encodes a protein of 201 amino acids containing four EF hand motifs. Many cis-acting elements related to plant stress and hormone response appear in the promoter regions of *SlCML39*. *SlCML39* is mainly expressed in the root, stem, and leaf and can be regulated by HT, cold, drought, and salt stresses as well as ABA and H_2_O_2_. Furthermore, heterologous overexpression of *SlCML39* reduces HT tolerance in *Arabidopsis thaliana* at the germination and seedling growth stages. To better understand the molecular mechanism of *SlCML39*, the downstream gene network regulated by *SlCML39* under HT was analyzed by RNA-Seq. Interestingly, we found that many genes involved in stress responses as well as ABA signal pathway are down-regulated in the transgenic seedlings under HT stress, such as *KIN1*, *RD29B*, *RD26*, and *MAP3K18*. Collectively, these data indicate that *SlCML39* acts as an important negative regulator in response to HT stress, which might be mediated by the ABA signal pathway.

## 1. Introduction

Calcium (Ca^2+^) is a key secondary messenger in eukaryotic cells which is involved in plant growth, development, and stress responses [1]. The Ca^2+^ signal is recognized by unique Ca^2+^ sensors, including calmodulin (CaM), calmodulin-like protein (CML), calcineurin B-like (CBL), and calcium-dependent protein kinase (CDPK) [1,2]. All these proteins have a conserved Ca^2+^ binding motif—“EF hand”—which binds to Ca^2+^ to cause conformational changes, followed by changes in activity to regulate downstream targets, thereby transmitting Ca^2+^ signals [3]. CaM is the most widely studied Ca^2+^ sensor. CMLs are closely related to CaMs, but their appearance is different from CaM, which has four EF hands. CMLs have one to six EF hands [4]. According to the existing genome database, many CMLs are identified in plants [5], such as 50 CMLs in *Arabidopsis thaliana* [6], 46 in *Medicago truncatula* [2], and 168 in *Brassica napus* [7]. Despite so many CML genes, the functions of most plant CMLs are not well studied [8].

To date, CML genes have been found to be involved in plant growth, development, cell metabolism, and stress tolerance. *AtCML24* and *AtCML25* mutants strongly affect Arabidopsis pollen germination and tube growth [9,10]. AtCML39 participates in light signal transduction and promotes the establishment of Arabidopsis seedlings [11]. AtCML42 transduces Ca^2+^ signal downstream by interacting with kinesin-interacting Ca^2+^-binding protein (KIC), then regulates the cell branches of hair [12] and also coordinates responses to *Spodoptera herbivory* [13]. CML41 regulates plasmodesmatal closure during plant immune responses [14]. CML36 regulates the activity of the Ca^2+^ ATPase ACA8 [15]. *AtCML9* knockout mutants exhibit stronger salt and drought tolerance through high accumulation of amino acids [16]. In Arabidopsis, a new rice CML gene *OsMSR2* endows plants with drought and salt tolerance through an ABA-mediated pathway [17]. *GsCML27* is involved in plant responses to bicarbonate, salt, and osmotic stresses [18]. CML20 negatively modulates ABA signals in guard cells and drought tolerance [19]. *MpCML40* overexpression strongly increases Arabidopsis salt tolerance [20]. In all, these studies suggest that CMLs likely interpret Ca^2+^ signals during development and stress responses.

To date, CML function analyses mainly come from Arabidopsis. In tomato, genome-wide identification and characterization of CML genes in two genetic backgrounds (*Solanum lycopersicum* and *Solanum pennellii*) were performed [21,22]. A total of 45 and 52 CML genes were identified in *Solanum pennellii* and *Solanum lycopersicum*, respectively. So far, however, there are only three reports on tomato CML function. A tomato CML (APR134) is involved in plant-*Pseudomonas syringae* interactions [23]. *ShCML44* overexpression improves tomato tolerance to cold, drought, and salinity stress [21]. Recently, SlCML37 has been shown to interact with proteasome maturation factor 1 (SlUMP1) to improve chill tolerance of tomato fruits [8]. The functions of other CMLs in tomato still need to be improved.

In a previous report, we found that a tomato (*Solanum lycopersicum*) CML gene (Solyc11g071740) was significantly induced by high temperature (HT) at transcription level using RNA-seq analysis of HT-responsive genes in tomato leaves [24]. However, the function of this gene responding to HT is unknown. In this study, the CML gene, named *SlCML39*, was characterized. *SlCML39* was mainly expressed in the root, stem, and leaf and could be regulated by different stresses. Moreover, heterologous overexpression of *SlCML39* remarkably reduced Arabidopsis tolerance to HT stress. The transcriptome by RNA-seq analysis further showed that many genes related with stress response as well as the ABA signal pathway were down-regulated in the transgenic seedlings under HT stress. Taken together, our results indicate that as an important negative regulator, *SlCML39* plays a role in HT stress response, which might be mediated by the ABA signal pathway.

## 2. Results

### 2.1. SlCML39 Is Attractive Enough under HT Stress

**Figure 1:**
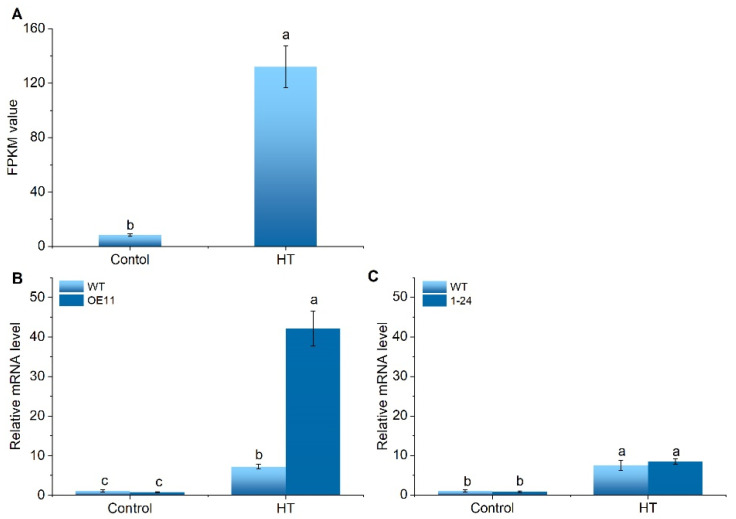
High temperature-induced *SlCML39* expression is regulated by *SlMPK1* under high temperature (HT). (**A**) The transcript level of *SlCML39* was induced by HT using RNA-seq analysis of HT-responsive genes in tomato leaves [24]. (**B**) qRT-PCR analysis of *SlCML39* expression in wild-type (WT) and *SlMPK1*-overexpressing line OE11 under HT. (**C**) qRT-PCR analysis of *SlCML39* expression in WT and *SlMPK1*-RNA interference line 1–24 under HT. The *UBI* gene was used as an internal standard. Total RNAs were extracted from WT, OE11, or 1–24 leaves under control (25 °C) and HT (42 °C) for 4 h. The bar represents the mean ± SD values of three replicates (*n* = 3). According to the LSD test, the statistical differences between samples are marked with different letters (*p* < 0.05).

Previously, we found that a tomato (*Solanum lycopersicum*) CML gene (Solyc11g071740) was significantly induced by HT at the transcription level using RNA-seq analysis of tomato HT-responsive genes [24]. Munir et al. [25] identified 52 CML genes in tomato (*Solanum lycopersicum*) using genome analysis and Solyc11g071740 was named as SlCML39. Therefore, we continue to use this name here. Interestingly, HT-induced *SlCML39* expression was mediated by SlMPK1 (Figure 1). Our previous data have shown that tomato SlMPK1 (mitogen-activated protein kinase 1) is a negative regulator in HT tolerance [26]. Here, HT-induced *SlCML39* expression was further increased in *SlMPK1*-overexpressing plants, but this inductive effect disappeared in *SlMPK1*-RNA interference plants under HT stress (Figure 1B,C), suggesting that *SlCML39* functions downstream of SlMPK1, and that SlMPK1 positively regulates the expression of *SlCML39* under HT stress. The discovery of *SlCML39* is exciting because its function can be studied to further elaborate the response mechanism of SlMPK1 to HT stress.

### 2.2. Characterization of SlCML39

**Figure 2:**
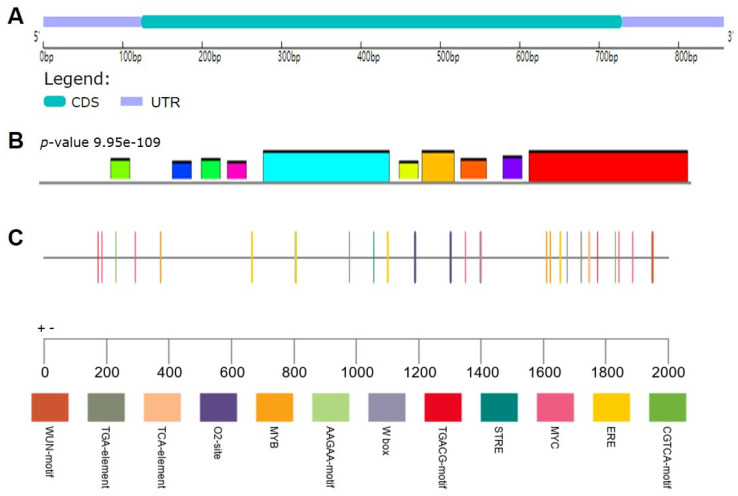
Characters of *SlCML39*. (**A**) *SlCML39* in the tomato genome. The *SlCML39* gene structure was visualized using GSDS 2.0. (**B**) The SlCML39 motif was analyzed using the MEME web server. (**C**) Cis-element analysis of *SlCML39* promoter. The cis-element of *SlCML39* promoter was analyzed by PlantCARE database. All elements are shown in Supplementary Table S1.

The CDS of *SlCML39* contains 606 bp open reading frame (ORF), which is on chromosome 11 without introns (Figure 2A). The cDNA encodes a protein of 201 amino acids, of which the predicted molecular mass is 22,118.77 Da and the theoretical pI is 4.79. Ten conserved motifs were predicted using the MEME tool (Figure 2B). To investigate the potential regulatory functions of *SlCML39*, the cis regulatory elements in the 2000 bp fragment upstream of the Transcription Start Site (TSS) were analyzed using the PlantCARE tool (Figure 2C, Table S2). The results showed that in addition to the core cis-elements such as TATA box and CAAT box, many types of cis-elements were found, including plant growth regulation, stress response, hormone response, and light response elements (Figure 2C, Table S2). For stress-responsive cis-elements, MYB and MYC were the most abundant elements. ARE (anaerobic response element), STRE (stress-response promoter element), as-1 (oxidative stress-responsive element), WUN-motif (wound-responsive element), and W-box (WRKY binding sites) were also present. For hormone-responsive cis-elements, four MeJA-responsive elements (CGTCA-motif and TGACG-motif), four estrogen response elements (ERE), one SA-responsive element (TCA-element), two GA-responsive elements (TATC-box, GARE-moti), and one auxin-responsive element (TGA-element) were detected in the promoter of *SlCML39*. The results here suggest that *SlCML39* might play a role in the transcriptional control of tomato growth, stress, and hormone responses.

**Figure 3:**
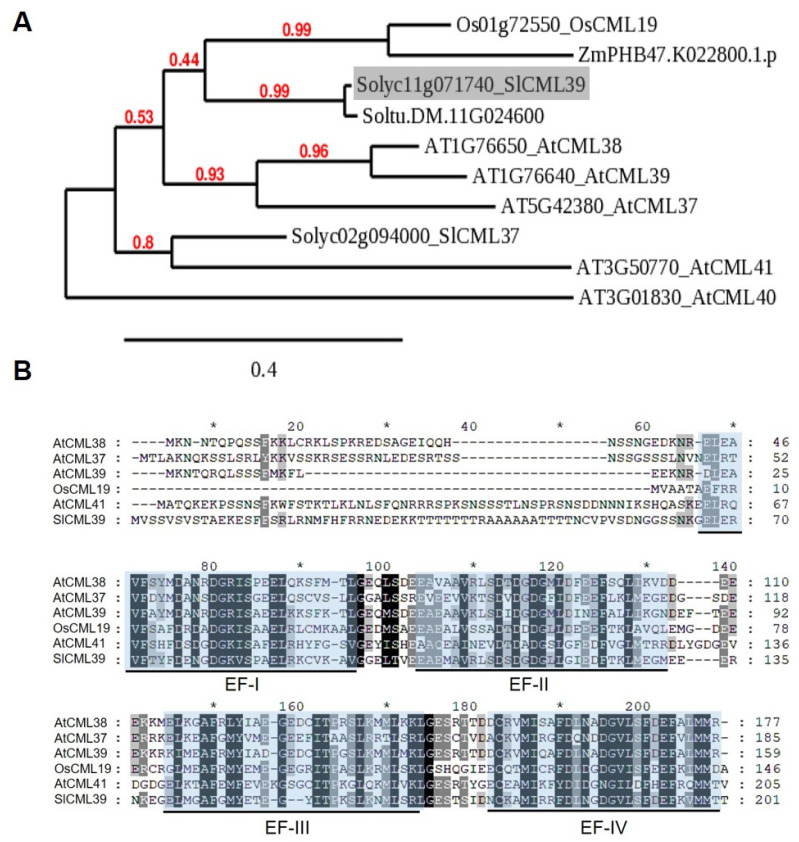
Phylogenetic tree and multiple sequence alignment of SlCML39 and its closely related CML proteins from other plant species. (**A**) The phylogenetic tree was constructed using MUSCLE/PhyML programs. The following protein sequences are included in the analysis: Arabidopsis (AT5G42380, AtCML37; AT1G76650, AtCML38; AT1G76640, AtCML39; AT3G01830, AtCML40; AT3G50770, AtCML41); *Oryza sativa* (Os01G72550, OsCML19); *Zea mays* (ZmPHB47.K022800.1.p); *Solanum lycopersicum* (Solyc11g071740, SlCML39); *Solanum tuberosum* (Soltu.DM.11G024600). SlCML39 is marked in gray. (**B**) Multiple sequence alignment of SlCML39 sequence with homologous CMLs from Arabidopsis and rice. Sequences were aligned by MUSCLE and shown by GeneDoc software. The EF hand motifs are underlined and the highly conserved amino acids in EF hand motifs are highlighted by the blue color.

To characterize the function of *SlCML39*, a phylogenetic tree was constructed according to the amino acid sequence of SlCML39 and all *Arabidopsis thaliana* AtCMLs (Figure S1), or some other plant species using MUSCLE/PhyML/TreeDyn programs (Figure 3A). The phylogenetic tree revealed that SlCML39 closely clustered with Soltu.DM.11G024600, which is because both tomato and potato belong to Solanaceae, so the homologous genes have the highest similarity. In addition to this protein, SlCML39 is also similar to some other members of the subfamily, including AtCML37, AtCML38, AtCML39, OSCML19, and ZmPHB47.K022800.1.p, which indicate that SlCML39 might have a similar function with these proteins. Recently, SlCML37 confers improved tolerance to tomato fruit chilling stress [8]. However, SlCML39 and SlCML37 are not the same protein (Figure 3A). A comparison of the protein sequences between SlCML39 and five homology proteins of other plants is presented in Figure 3B. SlCML39 shares a high degree of similarity to OsCML19 (56.74%), AtCML37 (56.03%), AtCML38 (54.48%), AtCML39 (54.031%), and AtCML41 (41.14%). Based on the InterPro protein families database (EF hand domain IPR002048) and Zhang et al.’s [20] analysis, the conservative functional domains identified in these CMLs are Ca^2+^-binding EF hands.

### 2.3. Expression Profiles of SlCML39

**Figure 4:**
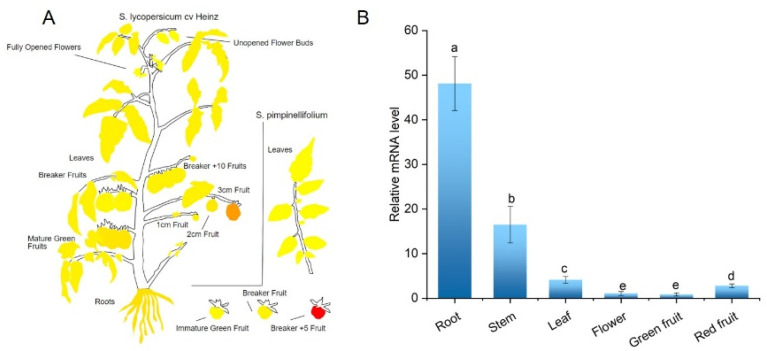
Expression levels of *SlCML39* in different organs. (**A**) ePlant of microarray data of *SlCML39* expression from bar.utoronto.ca. (**B**) The organ-specific expression of *SlCML39* was detected in the root, stem, leaf, flower, green fruit, and red fruit by qRT-PCR. Total RNA was extracted from different organs. The *UBI* gene was used as an internal control. The bar represents the mean ± SD values of three replicates (*n* = 3). According to the LSD test, the statistical differences between samples are marked with different letters (*p* < 0.05).

To study the spatial expression pattern of *SlCML39* in tomato, the ePLANT data for *SlCML39* expression was obtained from the tomato ePLANT browser (bar.utoronto.ca). Unfortunately, the expression of chip data was low and not accurate enough. Therefore, *SlCML39* expression levels in different organs were determined by qRT-PCR. The results displayed that *SlCML39* was expressed in most of the organs. The expression of *SlCML39* was the highest in root, followed by stem, leaf, and red fruit (Figure 4), indicating the organ specificity of *SlCML39* expression in tomato.

**Figure 5:**
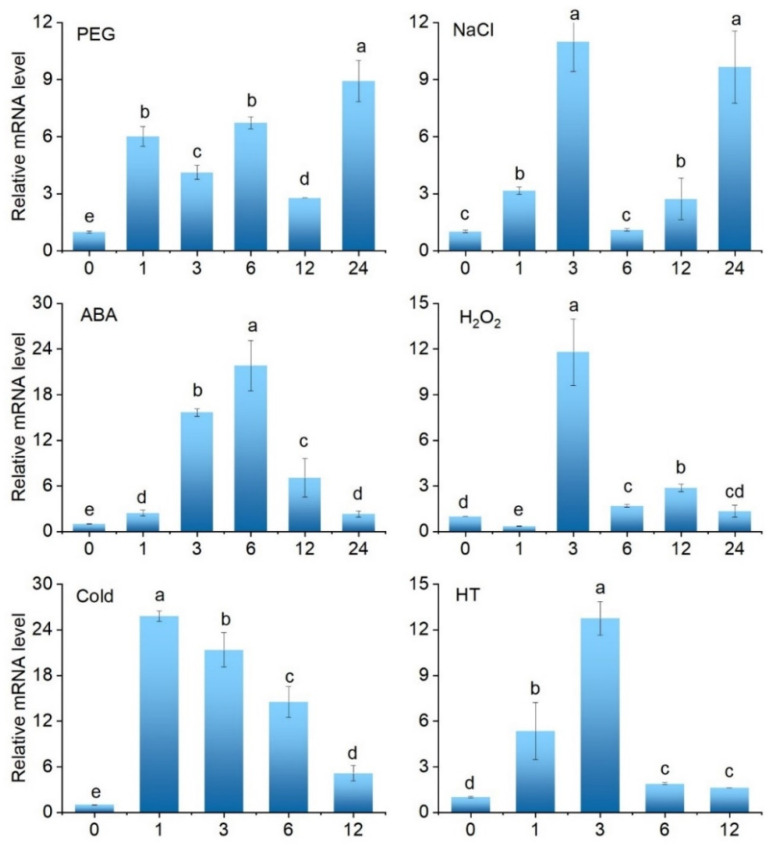
Expression levels of *SlCML39* under different treatments. Tomato seedlings were treated with H_2_O, 20%PEG6000, 100 mM NaCl, 100 uM ABA, 10 mM H_2_O_2_, cold (4 °C), or high-temperature conditions (HT, 42 °C) for 1, 3, 6, 12, and/or 24 h. Total RNA was extracted from leaves and relative mRNA levels were determined by qRT-PCR using *UBI* gene as an internal control. The fold change was normalized relative to the level of H_2_O treatment. The bar represents the mean ± SD values of three replicates (*n* = 3). According to the LSD test, the statistical differences between samples are marked with different letters (*p* < 0.05).

To explore the potential roles of *SlCML39* in stress responses, we then performed an analysis of the expression pattern of *SlCML39* under drought, salt, HT, cold, ABA, and H_2_O_2_ (Figure 5). Under 20% PEG6000 treatment, the expressions of *SlCML39* observably increased at different time points and reached the highest levels at 24 h; moreover, 100 mM NaCl stress could significantly induce *SlCML39* expression at the different time points, except for 6 h. After ABA treatment, the expression of *SlCML39* reached the highest levels at 6 h, then showed a gradual reduction. The *SlCML39* expression level was induced by 10 mM H_2_O_2_ at 3, 6, and 12 h, with about 12-fold accumulated transcripts at 3 h. The expression of *SlCML39* increased rapidly, reached the highest levels at 1 h, then showed a gradual reduction under cold stress (4 °C); HT (42 °C) significantly induced *SlCML39* expression, reaching the maximum at 3 h. These results indicate that *SlCML39* might be involved in response to multiple abiotic stresses (Figure 5).

### 2.4. SlCML39 Negatively Regulated Seed Germination in Arabidopsis under HT Stress

**Figure 6:**
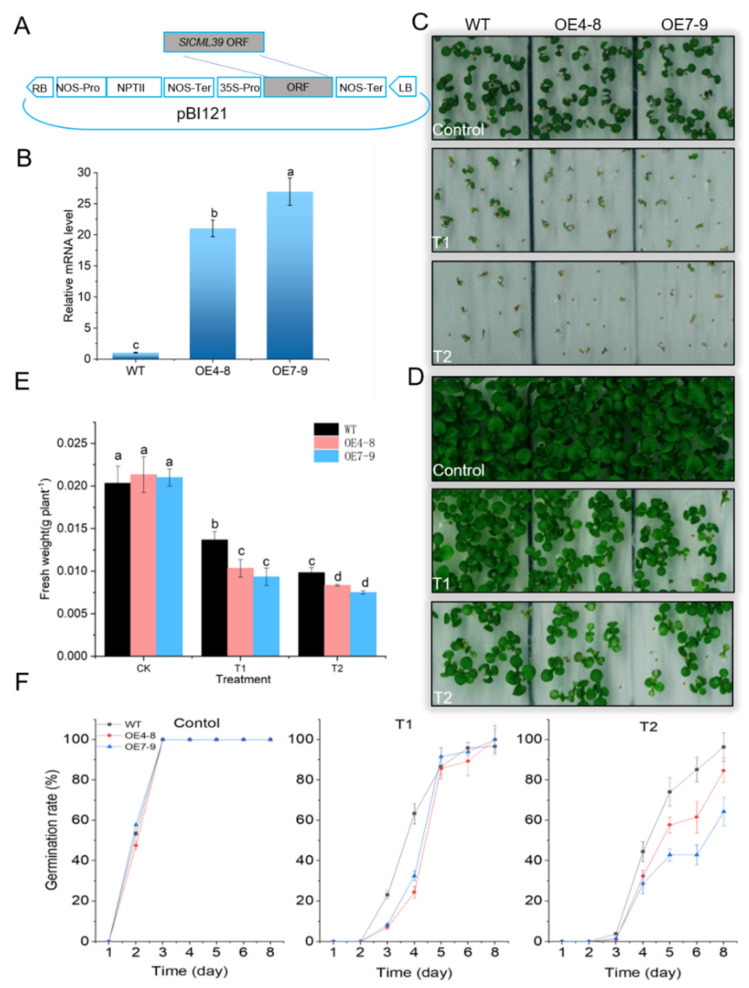
Overexpression of SlCML39 increases the sensitivity of Arabidopsis thaliana seed germination to high temperature (HT). (**A**) Schematic diagram of the SlCML39 expression vector. The coding region of SlCML39 was cloned in the expression vector pBI121. (**B**) The mRNA levels of SlCML39 in WT and two SlCML39-overexpressing transgenic lines, OE4-8 and OE7-9, using qRT-PCR. ACTIN gene was used as an internal control. (**C**) Seed germination of WT and SlCML39-overexpressing lines on 1/2 MS medium after HT. Dry seeds were immersed at 52 °C for 15 (T1) and 25 min (T2), then sterilized on the surface before sowing. The germination rate within 8 days was calculated. The phenotype under light for 4 days is shown. (**D**) Post-germinated growth of WT and SlCML39-overexpressing lines on 1/2 MS medium after HT for 14 days. (**E**) The fresh weight of (**D**). (**F**) The germination rates of (**C**). The bar represents the mean ± SD values of three replicates (*n* = 3). According to the LSD test, the statistical differences between samples are marked with different letters (*p* < 0.05).

*SlCML39* has a higher expression level at the transcription under HT stress (Figure 1 and Figure 5). HT-induced *SlCML39* expression is regulated by SlMPK1 (Figure 1), which has been found involved in thermotolerance [26]. We speculated that *SlCML39* might be involved in HT response. To prove this, the transgenic Arabidopsis plants overexpressing SlCML39 were obtained (Figure 6A). Two homozygous lines (OE4-8, OE7-9) were obtained and used to evaluate thermotolerance. The expression level of *SlCML39* in OE4-8 and OE7-9 was verified by qRT-PCR analysis (Figure 6B). First, we conducted a plate germination test to determine the HT tolerance of Arabidopsis lines OE4-8 and OE7-9 and WT (Figure 6C–F). Seeds of Arabidopsis were immersed at 52 °C for 15 or 25 min, then sown on 1/2 MS medium. The germination rate and posted-growth phenotype were observed within 8 days. Under normal conditions (seeds without HT pretreatment), OE4-8 and OE7-9 plants showed similar germination and post-geminated growth with WT, indicating that *SlCML39* does not affect seed germination (Figure 6C,F) and early seedling growth (Figure 6D,E). After HT pretreatment, however, OE4-8 and OE7-9 plants displayed lower germination rates, lower biomass of post-germinated seedlings, and fewer seedlings with open and green leaves than WT. In all, during imbibition, transgenic *SlCML39*-overexpressing seeds exhibited a thermosensitive phenotype.

### 2.5. SlCML39 Overexpression Decreased Arabidopsis Seedling Tolerance to HT Stress

**Figure 7:**
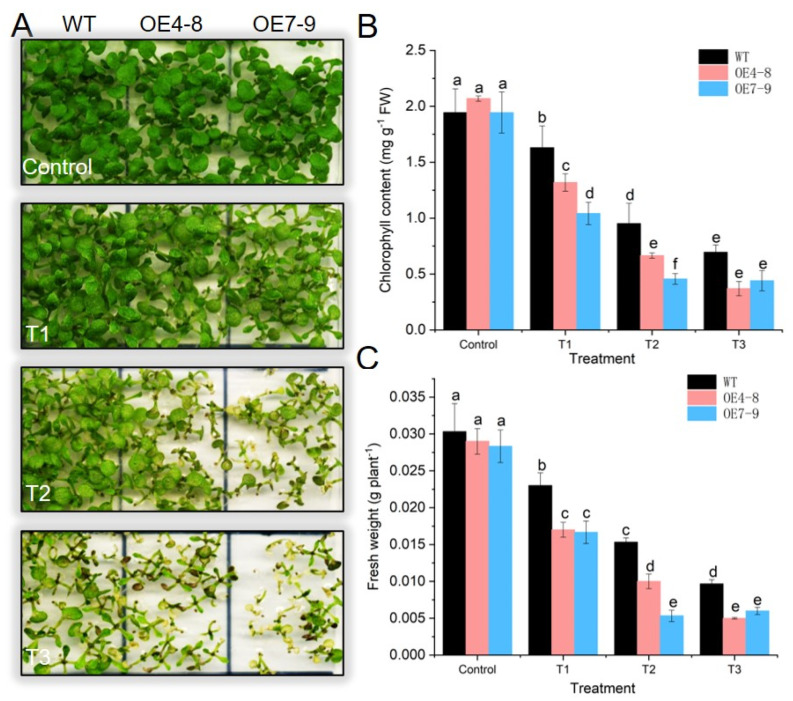
Overexpression of *SlCML39* increases the sensitivity of seedling growth to high temperature (HT). (**A**) Phenotype of seedling growth of *SlCML39*-overexpressing lines (OE4-8 and OE7-9) after HT. After germination on 1/2 MS medium for 7 days, the seedlings on plates were exposed to 45 °C for 0 min (control), 45 min (T1), 60 min (T2), and 90 min (T3), followed by recovery under standard conditions (23 °C) for 7 days. (**B**) Chlorophyll content of (**A**). (**C**) Plant fresh weight of (**A**). The bar represents the mean ± SD values of three replicates (*n* = 3). According to the LSD test, the statistical differences between samples are marked with different letters (*p* < 0.05).

HT-induced inhibition of seedling growth assay has been used to identify plant HT tolerance [26]. Consistent with the seed germination type, there was no obvious difference between WT and transgenic line OE4-8 and OE7-9 plants during the growth stage under the standard conditions (Figure 7), suggesting that *SlCML39* does not affect seedling development. After exposure to HT stress, all plants showed burning symptoms caused by HT. However, *SlCML39-*overexpressing plants had a more serious HT damage phenotype, lower chlorophyll content, and lower fresh weight when compared with WT (Figure 7). With the extension of HT treatment time, the damage phenotype caused by HT aggravated, which was more serious in transgenic plants than in WT. Together, the results demonstrated that *SlCML*39 plays a negative role in HT tolerance.

### 2.6. RNA-Seq of SlCML39-Regulating Gene Network under HT Stress

In order to fully provide the regulatory network of *SlCML39* in HT response in Arabidopsis and gain insight into the mechanism of *SlCML39*, we conducted an RNA-seq analysis to screen DEGs-regulated by *SlCML39*. According to Lu’s experimental design of analyzing HT response by RNA-seq [27], we performed an RNA-seq analysis of OE7-9 and WT under HT stress using BGISEQ-500 platform [28]. Six cDNA libraries (three replicates per library) were set up from OE7-9 and WT plants under HT stress, and the boxplots revealed that the overall range and distribution of FPKM values of these samples are consistent (Figure S2), indicating that the RNA-seq data in this study have reproducibility, high quality, and reliability. Additionally, we analyzed the expression profiles among the two groups in the pairwise comparisons. DEGs were analyzed with a 2.0-fold change as a selection threshold. Gene expressions with a ≥2.0-fold or ≤0.5-fold change were recorded for significant up- or down-regulation, respectively; an adjusted *p*-value (padj) ≤ 0.05 was applied to both screenings.

**Figure 8:**
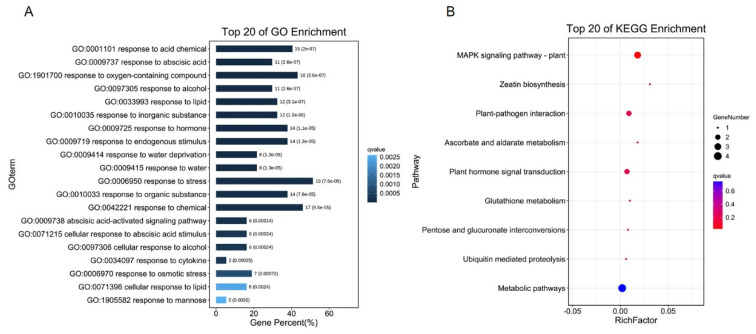
GO term and KEGG enrichment analyses of DEGs. (**A**) Top of 20 GO term enrichment. The vertical coordinates are the enriched GO terms, and the horizontal coordinates are the numbers of the DEGs. (**B**) KEGG enrichment analysis of DEGs. The vertical coordinates are the enriched pathways, and the horizontal coordinates are the rich factors. The size of each point represents the number of DEGs in the pathway, and the point color represents the *q*-value.

Through rigorous screening for three biological repetitions, we screened a total of 38 DEGs (Table 1), of which 28 were down-regulated and 10 were up-regulated in *SlCML39-*overexpressing line OE7-9. According to GO annotation, all DEGs are divided into 34 groups, including 19 biological processes, 6 cellular components, and 9 molecular functions (Figure S2B). In biological processes, the DEGs are mainly involved in cellular processes, metabolic processes, stimulus response, biological regulation, and the regulation of biological processes. The 20 top GO enrichment shows that a large number of stress-responsive DEGs are modulated by *SlCML39*, such as “response to stimulation (19)”, “response to hormone (11)”, and “response to ABA (11)” (Figure 8 and Figure S2C). In terms of molecular function, there are two categories: “binding” and “catalytic activity”. To screen the pathways regulated by *SlCML39*, the KEGG pathway annotation was performed, and MAPK signal pathway is the only pathway with a *q*-value < 0.05. To further confirm the results, qRT-PCR analysis of six DEGs was used to validate the data. Six genes displayed similar expression trends at the transcript level (Figure S3). The results indicate that *SlCML39*-regulated DEGs might be responsible for the decreased HT tolerance in *SlCML39*-overexpressing plants.

### 2.7. SlCML39 Negatively Regulates Stress/ABA-Responsive Genes under HT Stress

Because 38 DEGs were mainly clustered into “response to stress” and “response to ABA” and *SlCML39* expression was also induced by HT, we screened the genes of HT response and ABA response. Chao et al. [29] mapped heat-responsive genes to an ABA-responsive gene pool to identify whether the heat-responsive genes were also ABA-responsive genes. In this study, although these genes were not clustered into the heat shock response, we mapped 38 HT-responsive DEGs list to three HT-responsive gene pools [30,31,32] and two ABA-responsive gene pools [33,34] to discern whether these DEGs were HT or ABA-responsive genes. Combined with GO analysis and Arabidopsis eFP Browse database, 33 and 22 DEGs were identified to be involved in HT and ABA response, respectively (Table 1 and Table S3). Moreover, 21 of the 33 DEGs (63.6%) that responded to HT were able to respond to ABA (Table 1). Interestingly, the 28 down-regulated DEGs in this study were compared to the study in 2016 [33] and it was found that 16 of them were ABA-responsive genes such as *RD29B*, *DAA1*, *RD26*, *FAMT*, *SAG113*, *EDL3*, *SIS*, *MAPKKK18*, *KIN1*, and *DIL4* (Table S4). Many DEGs have been found in HT tolerance. For example, the transcription levels of *RD29B*, *RD26*, *FAMT*, *SAG113*, *EDL3*, *KIN1*, and *DIL4* were induced by HT, of which the *RD26*, *SAG113*, *KIN1*, and DIL4 expressions in heat-sensitive *gcn5* Arabidopsis were lower than that in WT [31]. Here, the visualization of RNA-seq readings of these genes confirmed that the expressions of these genes are down-regulated in *SlCML39*-overexpressing plants compared to WT after application of HT. *RD29B*, *RD26*, *SAGT1*, *SAG113*, *KIN1*, *DIL4*, and *AFP2* are ABA-responsive genes, which play an important role in ABA signal and stress responses [35,36]. ABA is also involved in acquired thermotolerance [37,38]. Except for *AFP2*, the expression levels of these DEGs under HT stress were also decreased in *SlCML39*-overexpressing plants compared with WT. In all, there were 21 DEGs responding to HT and ABA simultaneously, indicating that the negative regulation of HT tolerance by *SlCML39* might be mediated by ABA signal. Additionally, three DEGs involved in the MAPK signal pathway (*HAI1*, *MAPKKK18*, and *MKK5*) were down-regulated in *SlCML39* overexpression plants. MAPK cascade is involved in response to HT stress [26,39]. ROS-induced redox signal acts as an important regulator of various stress responses [40]. Interestingly, the expression levels of *ATMDAR3*, *ATMSRB7*, and *GSTF7* decreased in the *SlCML39*-overexpressing plants under HT compared with WT (Table 1).

To evaluating the reliability of results of RNA-Seq, the relative expressions of six typical stress/ABA-responsive DEGs in the *SlCML39* overexpression plant and WT treated with or without HT were validated by qRT-PCR (Figure S3). The expression pattern of the selected stress-related genes by qRT-PCR mostly agreed with RNA-Seq data, although the absolute fold changes of the two methods were different, indicating that the results obtained by RNA-seq analysis are credible.

### 2.8. SlCML39 Is Involved in ABA-Mediated Seed Germination

**Figure 9:**
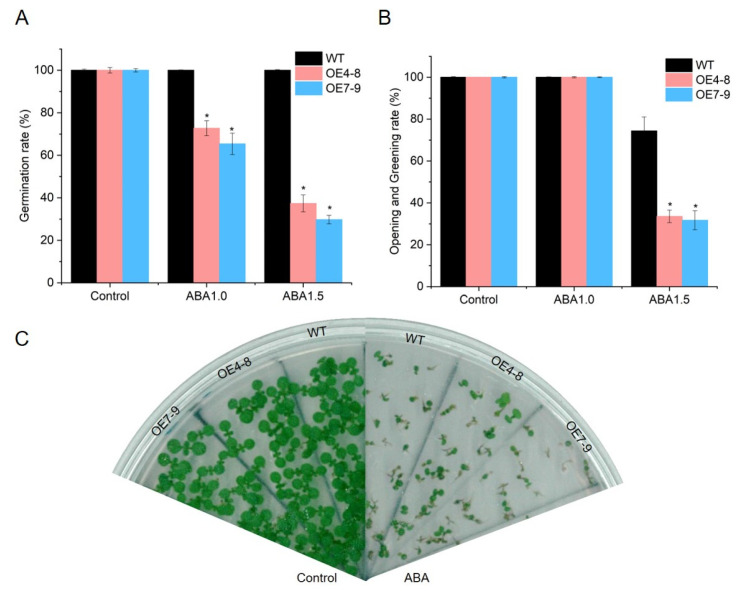
Overexpression of *SlCML39* increases *Arabidopsis thaliana* sensitivity to ABA. (**A**) The germination rate of *SlCML39-*overexpressing lines (OE4-8 and OE7-9) and wild-type (WT) Arabidopsis seeds on 1/2 MS medium supplementing 1.0 and 1.5 µm ABA. Germination was recorded daily up to 5 days. (**B**) Seedlings with open and green leaves were recorded at 10 days. (**C**) The photographs were taken at 10 days. The bar represents the mean ± SD values of three replicates (*n* = 3). * *p* < 0.05 by Student’s *t*-test.

To further confirm the role of *SlCML39* in ABA signal, we tested the ABA sensitivity of *SlCML39*-overexpressing plants (OE4-8 and OE7-9) relative to WT plants at the germination and post-germination stages under ABA treatment. *SlCML39* ectopic expression lines exhibited similar seed germination with WT. However, under 1.0 and 1.5 µm ABA treatment, transgenic lines displayed lower seed germination rates (Figure 9A) than WT. The higher the ABA concentration, the stronger the inhibition. The hypersensitivity of *SlCML39-*overexpressing plants to ABA was also observed in the post-germination stage. Under normal conditions, there was no significant difference in cotyledon opening rate and greening rate between transgenic plants and WT (Figure 9B,C). Under 1.0 µm ABA, there was no obvious difference. However, under 1.5 µm ABA supplement, the cotyledon opening and greening rates of OE4-8 and OE7-9 were inhibited more severely in transgenic lines than in WT (Figure 9B,C). The results demonstrated that *SlCML39* confers plants more sensitive to ABA-inhibited germination and post-geminated growth, suggesting that *SlCML39* is involved in the ABA signal.

## 3. Discussion

### 3.1. Homologous CMLs of SlCML39

The CMLs are a unique group of EF hand proteins in plants, which bind to Ca^2+^ and regulate downstream signal transduction [3]. In *Arabidopsis thaliana*, 50 AtCMLs were identified according to Arabidopsis genome sequence, which are divided into eight groups [6]. In this study, SlCML39 belongs to Ca^2+^-binding proteins with four EF hands, which are clustered in group IV of AtCMLs [6]. SlCML39 has the highest sequence similarity with AtCML37, AtCML38, and AtCML39. These three CMLs act as sensors in Ca^2+^-mediated growth, development, and stress response [41]. *AtCML37* loss-of-function mutants showed increased sensitivity to herbivores and decreased tolerance to drought stress [42]. CML38 is a core hypoxia response Ca^2+^ sensor protein and serves as a potential Ca^2+^ signal target during flooding stress response [43]. As an AtRALF1-interacting partner, CML38 participates in the inhibitory effect of AtRALF1 on root growth [44]. CML38 specifically interacts with PEP1 RECEPTOR 2 (PEPR2) and negatively regulates Arabidopsis root elongation under low nitrate [45]. As a Ca^2+^ sensor, CML39 is involved in the transduction of light signals that promote seedling establishment [11]. CML39 plays a key role in seed and fruit development and hormone-mediated seed dormancy [46]. Recently, it was found that SlCML37 interacts with proteasome maturation factor SlUMP1 and confers improved chilling tolerance in tomato fruit [8]. All these results imply that SlCML39 might have similar functions, which requires further functional studies.

### 3.2. SlCML39 Expression Is Induced by HT as well as Other Stresses

We speculated that *SlCML39* could function under HT stress because of its higher expression induced by HT (Figure 1). To date, there is no report on the functional research of CMLs in response to HT, while some CML genes in plants can respond to HT stress. It is reported that Arabidopsis *AtCML12* (*TCH3*) and *AtCML24* (*TCH2*) expressions are highly induced by heat stress [47]. *AtCML44* is strongly induced by heat [6]. Two *BnaCML44* genes (BnaA06g15280D and BnaC05g16860D) are enhanced by heat [7]. In this study, HT (42 °C) significantly induced *SlCML39* expression, reaching the maximum in 3 h (Figure 5). In addition, *SlCML39* expression was regulated by drought, salt, HT, and cold stresses, as well as exogenous ABA and H_2_O_2_, similar to the previous studies. For example, the expressions of *AtCML37*, *AtCML38*, and *AtCML39* genes are induced by a variety of stimuli such as salt and drought stress [41]. *AtCML9* expression in young seedlings is rapidly induced by cold, salinity, and dehydration treatments [16]. Expression of 32 CML genes in wild-growing grapevine shows that it can respond to drought, salt, heat, and cold [48]. Through analysis of upstream promoter sequences with the promoter tool PlantCARE, a variety of different boxes correlated with responses to miscellaneous abiotic stress were found in the *SlCML39* promoters (Figure 2C). A variety of stress and hormone response elements were found by promoter element analysis, but there were no heat shock elements (HSE) or ABA-responsive elements (ABRE) (Figure 2C). Heat-induced genes may not always require HSE. Several Dof sequences, a W-box, and an MYC recognition site are present in the heat-responsive region of the *MsDREB2C* gene [49]. In general, ABRE is necessary for ABA-induced genes. However, other cis-elements that mediate ABA regulated transcription are also known. For example, MYB and MYC binding sites (without any G-ABRE or coupling elements) in the promoter region of *RD22* gene are crucial for ABA-dependent drought-induced gene expression [50]. In all, in addition to HT, other stresses could also regulate *SlCML39* expression. *SlCML39* can be considered as a stress-responsive gene because its expression rapidly changes in response to these stresses. In this study, we focus our research on the function of *SlCML39* in HT response.

### 3.3. SlCML39 Decreased Plant Thermotolerance

To explore the roles of *SlCML39* in tomato under HT stress, *SlCML39* was heterologously expressed in Arabidopsis. The transgenic plants overexpressing *SlCML39* displayed lower seed germination rates, fewer seedlings with open and green leaves, lower biomass of post-germinated seedlings than WT, and worse seedling growth than WT after HT stress (Figure 6 and Figure 7). It is indicated that *SlCML39* plays a negative role in regulating plant thermotolerance. So far, most studies on CMLs show a positive regulation of stress [8,16,17,20,21]. For example, *OsMSR2* is involved in drought and salt tolerance [17]. Overexpression of *MpCML40* enhances salt tolerance of Arabidopsis [20]. Of course, earlier reports of many systems revealed their negative impacts on plant defense. *CML24*-underexpressing transgenics enhance Arabidopsis tolerance to various salts such as CoCl_2_, molybdic acid, ZnSO_4_, and MgCl_2_ [51]. *CML9* is considered as a negative regulator of ABA-dependent salt and drought tolerance [16]. *CML42* has a negative effect on ABA biosynthesis upon drought stress [13]. *GsCML27* overexpression decreased the salt and osmotic tolerance of Arabidopsis [18]. *AtCML20* is a negative regulator in Arabidopsis of ABA-induced stomatal movement and drought tolerance [19]. One explanation for these regulatory mechanisms is the specificity and complexity of CML functions in virous stress signal pathways. However, different regulatory pathways may have common components as crosstalk nodes. Among these regulatory mechanisms, one of the most striking is the CML-mediated ABA signal [13,16,17,19]. Interestingly, these HT-intolerant phenotypes observed in the *SlCML39-*overexpressing plants are attributed to its function of regulating the expression levels of ABA- and HT-responsive genes (Table 1).

### 3.4. SlCML39 Is Involved in ABA-Regulated Germination

It is well known that Ca^2+^ is a component of the ABA signal pathway and some CML proteins have been proved to be involved in this pathway [17,46,51]. It is well established that ABA can maintain seed dormancy, arrest seed germination, and prevent seedling growth, and HT can induce ABA biosynthesis and trigger ABA-mediated signal pathways [29,37]. In the current study, HT exerted its inhibitory effect on seed germination and post-germinated growth of WT and transgenic plants mostly through ABA production. Under HT stress, the ABA level was up-regulated (Figure S4). However, there was no significant change in the ABA content of transgenic plants compared with WT under HT stress (Figure S4). On the contrary, there was increased sensitivity of the *SlCML39*-overrexpressing transgenics to HT. These results suggest that *SlCML39* is not involved in ABA biosynthesis, but rather in the ABA signal pathway under HT. Furthermore, the seed germination experiment regulated by ABA further confirmed this assumption. Previous studies have shown that CMLs function in ABA-regulated seed germination. For instance, during the seed germination and post-germination stages, transgenic *OsMSR2* overexpression plants exhibit hypersensitivity to ABA [17]. In this work, Arabidopsis-overexpressing *SlCML39* increased sensitivity to exogenous ABA (Figure 9). The seeds of transgenics and WT germinated fully under normal conditions. With the increase in ABA content, the germination rate and cotyledon greening of transgenics seeds were more severely reduced by ABA (Figure 9). This result indicates that *SlCML38* acts downstream of the ABA signal, resulting in ABA hypersensitivity during seed germination. Previously, *CML24* may act on the downstream signal of ABA perception, perhaps mediating the responses of cells to ABA-induced Ca^2+^ fluctuations to delay seed germination and seedling growth [51]. The *atcml9* mutant germination is hypersensitive to exogenous ABA and *AtCML9* does not play a key role in ABA biosynthesis, but rather in the ABA signal pathway as a negative regulator [16]. The sensitivity of seeds to ABA inhibition of germination is significantly reduced in Arabidopsis *cml39* mutant and mutant plants are more likely to be affected than endogenous ABA content [52]. Together, the existing evidence shows that plant CMLs play special functions downstream of the ABA signal. 

### 3.5. SlCML39 Regulates Stress/ABA-Responsive Genes under HT

ABA is a key hormone regulating plant tolerance to abiotic stresses, including temperature change and drought [52]. Application of ABA enhances plant HT tolerance, and the ABA-mediated signal is involved in acquired HT tolerance, as well [37]. Heat stress induces many ABA-responsive genes [29]. To further study the molecular mechanism of the *SlCML39* negative regulation of HT tolerance, RNA-seq was used to identify the gene network. *SlCML39*-overexpressing plants showed alterations in the expression of several stress and ABA-responsive genes, many of which were associated with HT stress (Table 1 and Table S4). GO enrichment analyses revealed that many DEGs were significantly enriched in the ABA signal pathway and 17 of the 38 DEGs were able to respond to HT and ABA at the same time (Table 1), supporting the notion that Arabidopsis thermotolerance involves the induction of a subset of ABA-responsive genes. Here are two puzzling questions to answer. One is that ABA synthesis-related genes are not found in this study (Table 1). Combined with the above results of *SlCML39* involved in seed germination and ABA accumulation level, it is indicated that *SlCML39* contributes to HT response in conjunction with the downstream ABA signal, and a role of *SlCML39* in regulating stress/ABA-related genes is suggested. Another indication is that HT-related genes of heat shock proteins (HSPs) and heat shock factor (HSFs) are not present. Interestingly, heat stress-triggered hormone responses are independent of the HSP/HSF pathways, indicating the existence of specific links between heat shock perception and hormone responses [29]. Geng et al. [53] revealed that ER stress-related genes are associated with HT tolerance in *TabZIP60s* overexpressing Arabidopsis by RNA-sequencing analysis, and also few HSP/HSFs are found under HT stress. Our present results imply a specific link between *SlCML39* and ABA signal in response to HT stress.

**Figure 10:**
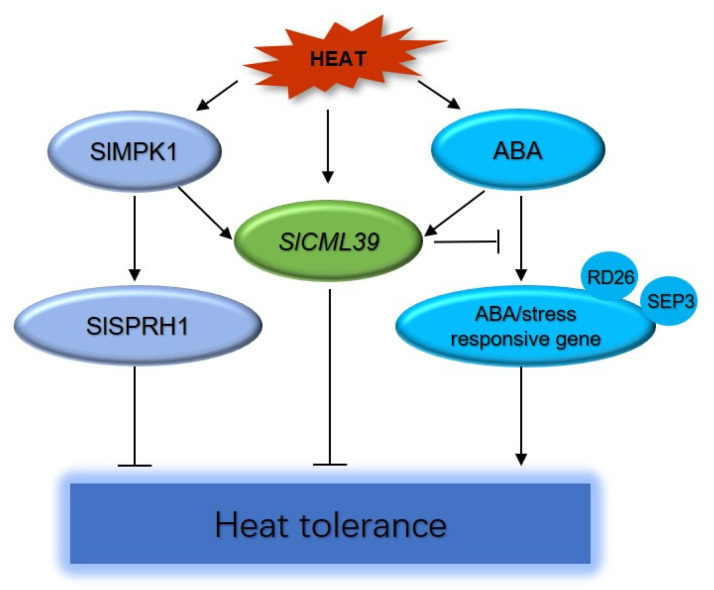
The model of SlCML39 as a hub connecting the ABA signal and ABA-mediated heat tolerance. Under heat stress, phosphorylation of the SlMPK1 activates downstream target proteins SlSPRH1, resulting in decreased thermotolerance [26]. Heat-induced *SlCML39* expression is mediated by SlMPK1, and is involved in negative regulation of heat tolerance. Under heat stress, *SlCML39* expression is induced, acts downstream of ABA signal, and inhibits the expressions of ABA/stress-responsive genes, resulting in decreased thermotolerance. The NAC transcription factor RD26/ANAC072 and MADs box transcription factor SEP3 are involved.

Previous studies have also proved the regulatory relationship between CML and ABA, mainly focusing on drought and salt, and only analyzed some response genes. The *atcml9* mutants alter the expression of some stress and ABA-responsive genes as well as defense-related genes [16]. Xu et al. [17] showed that Arabidopsis *OsMSR2* confers salt and drought tolerance, accompanied by changes in the expression of stress/ABA-responsive genes such as *RD29A*, *P5CS1*, and *ABI3*. In the *cml20* mutant, the expressions of stress genes including *MYB2*, *RAB18*, *ERD10*, *COR47*, and *RD29A* are up-regulated under drought and ABA treatment, suggesting that CML20 plays a role in ABA and drought stress signal pathways [19]. What attracts attention is that this study is the first to study the function of CMLs in HT stress, and proposes that *SlCML39*-mediated HT tolerance is related to ABA signal pathway. Here, several abiotic stress/ABA-responsive genes such as *KIN1*, *RD26*, and *RD29B* have reduced expression in *SlCML39-*overexpressing plants (Table 1). Additionally, many genes also play roles in stress tolerance. *AtSIS* is a salt-induced serine-rich gene and plays a role in salt tolerance, probably by increasing the protective effect of membranes [54]. Exposed to osmotic stress, high salinity, and ABA, *EDL3* transcript accumulates and plays an active regulatory role in ABA-dependent signal cascades, regulating germination induction, root growth, greening of etiolated seedlings, and anthocyanin accumulation under drought stress [55]. *DAA1* (At1g64110) encodes an AAA+-type ATPase and the response of *DAA1* gene knockout mutant to ABA, NaCl, and mannitol is impaired in germination and root growth, indicating stress sensitivity and the role of the *DAA1* gene in stress tolerance [56]. *OsRZFP34*, a homolog *AtRZFP34* (At5g22920) in rice, is markedly higher with HT and ABA at the mRNA level in transgenic rice, and *OsRZFP34* may increase stomatal opening and transpiration cooling function of rice under HT [57]. SGT1 is a co-chaperone of HSP90 and the response of plants to elevated ambient temperature requires an HSP90–SGT1 chaperone system [58]. Two transcription factors (TFs) were found to be involved in the SlCML39-mediated pathway, including RD26/ANAC072 and SEP3/AGL9 (Table 1, Figure 10). RD26 is a NAC TF and functions as a transcriptional activator in ABA-inducible gene expression under abiotic stress in plants [59]. SEP3 is a MADS-box TF and ABA was reported to upregulate the expressions of MADS-box genes. However, the direct relationship between SEP3 and ABA needs to be further studied.

KEGG enrichment analyses showed that three genes, *MAPKKK18*, *MKK5* and *SAG113*, were significantly enriched in the MAPK signal pathway (Figure 8, Table S3). *MKKK18* expression is modulated by ABA and MAPKKK18 positively regulates drought tolerance via downstream MPKK3 in Arabidopsis [60]. MKK5 is involved in AIK1-modulated ABA response through the MKK5-MPK6 kinase cascade and functions in the regulation of ABA on the primary root growth and stomatal response [61]. Inhibition of *MKK5* can reduce the transmission of ozone-induced signals to MPK3 and MPK6 and increase ozone sensitivity in Arabidopsis [62]. In the current study, the expressions of *MAPKKK18* and *MKK5* were induced by HT (Table 1). HT-induced *SlCML39* expression was further increased in SlMPK1-overexpressing plants, but this inductive effect disappeared in SlMPK1–RNA interference plants under HT stress (Figure 1). These results suggest that MAPK cascade is involved in *SlCML39*-regulating HT tolerance, but the specific cascade pathway still needs to be studied.

## 4. Materials and Methods

### 4.1. Plant Material Growth

Tomato (*Solanum lycopersicum* cv. “OFSN” background: wild-type (WT), *SlMPK1*-overexpressing line OE11, and *SlMPK1*-RNA interference line 1–24 [26]) and *Arabidopsis thaliana* (Columbia-0 ecotype background: WT and *SlCML39*-overexpressing lines (OE4-8 and OE7-9) were used in the present study. For tomato growth, the germinated seeds on the filter paper in the dark were planted in plastic pots containing soil mixture and grown at 25 °C/20 °C (day/night) and 14 h of light/10 h of dark with 70% relative humidity. At the five-leaf stage, robust seedlings with the same size were selected for the following treatments. For Arabidopsis growth, Arabidopsis seeds with or without HT stress were sterilized on the surface and planted on 1/2 Murashige and Skoog (MS) medium and grew under a 14 h of light/10 h of dark at 23 °C. Five-day-old Arabidopsis seedlings were used for HT treatment.

### 4.2. Expression Analysis of SlCML39

First, we clarified the SlMPK1-mediated expression of *SlCML39*. The *SlMPK1*-overexpressing line OE11 and *SlMPK1*-RNA interference line 1–24 were used in our previous report [26]. The four-week-old seedlings of WT, OE11, and 1–24 were treated with control (25 °C) and HT (42 °C) for 4 h. The leaves were collected for further RNA extraction.

Second, we clarified the organ-specific expression of *SlCML39*. The ePLANT data of *SlCML39* expression were obtained from bar.utoronto.ca (tomato eFP browser). RNA sequence expression data were obtained from different organs of *Solanum lycopersicum* cv. Heinz. qRT-PCR was used to detect the organ-specific expression of *SlCML39*. Total RNA was isolated from tomato root, stem, leaf, flower, green fruit, and red fruit.

The four weeks-old tomato seedlings were exposed to drought, salt, HT, cold, H_2_O_2_, and ABA treatments for 1, 3, 6, 12, and/or 24 h. For salt and drought stresses, the plants were immersed in 100 mM NaCl and 20% poly(ethylene glycol)-6000, respectively, for 1, 3, 6, 12, and 24 h. In terms of HT and cold stresses, the seedlings were exposed to HT (42 °C) and cold (4 °C), respectively, for 1, 3, 6, and 12 h. Then, 100 uM ABA and 10 mM H_2_O_2_ were sprayed on tomato leaves for ABA and H_2_O_2_ treatments, respectively, and the processing time lasted 24 h. The seedlings under normal conditions were used as control. The leaves were collected at the appointed time and preserved at −70 °C for further RNA extraction.

### 4.3. Bioinformatics Analysis of SlCML39

The location of *SlCML39* in chromosomes was checked in the Tomato ITAG4.0 genome database and the *SlCML39* gene structure was confirmed by NCBI and visualized by GSDS 2.0 (Gene Structure Display Server 2.0 (gao-lab.org), accessed on 1 August 2021). Online PlantCARE analyzed the cis-regulatory elements in the 2000 bp promoter region from Transcription Start Site (TSS) of *SlCML39* downloaded from ITAG4.0 (Bioinformatics and Systems Biology (ugent.be), accessed on 1 August 2021), and the cis-elements were analyzed by the PlantCARE database (https://solgenomics.net, accessed on 1 August 2021), which were visualized on the TBtool software. The MEME tool (MEME Suite (meme-suite.org), accessed on 1 August 2021) was used to predict and analyze the conserved motifs of tomato SlCML39. The EF hand motifs were analyzed according to Zhang et al. [20]. The PI and MW of SlCML39 protein were measured using the online ExPASy tool (https://web.expasy.org, accessed on 1 August 2021).

Sequence alignment of SlCML39 with homologous CMLs of Arabidopsis and rice were aligned using MUSCLE [63] and translated by GeneDoc software including Arabidopsis AT5G42380 (AtCML37), AT1G76650 (AtCML38), AT1G76640 (AtCML39), AT3G50770 (ATCML41); *Oryza sativa* Os01G72550 (OsCML19); and *Solanum lycopersicum* Solyc11g071740 (SlCML39). The phylogenetic tree was constructed using MUSCLE, PhyML, and TreeDyn at www.phylogeny.fr [63]. Along with the six proteins in the above sequence alignment, AT3G01830 (AtCML40), *Zea mays* (ZmPHB47.K022800.1.p), Solyc102g094000 (SlCML37), and *Solanum tuberosum* (Soltu.DM.11G024600) were also included.

### 4.4. Heterologous Expression of SlCML39 in Arabidopsis

To construct transgenic Arabidopsis lines overexpressing *SlCML39*, the full-length cDNA fragment of *SlCML39* was inserted in the binary vector pBI121 driven by CaMV 35S promoter. Arabidopsis transformation was carried out by the floral dip method. The T1 transformant was screened by detecting the (3:1) isolation rate of kanamycin resistance according to Ding et al. [26]. Two T2 generation homozygote plants (OE4-8 and OE7-9) were obtained, and the expression level of *SlCML39* was analyzed by qRT-PCR. The two lines were also used for the study.

### 4.5. HT Tolerance of Arabidopsis

For seed germination and post-germinated growth, dry seeds of WT and *SlCML39-*overexpressing line OE4-8 and OE7-9 were immersed at 52 °C for 15 (T1) and 25 min (T2) according to the method of Yokotani et al. [64]. After imbibition, seeds were sterilized on the surface before sowing on 1/2 MS medium. The germination rate and posted-growth phenotype were observed within 8 days of seed germination. For seedling growth, the plates containing 7-day-old seedlings were exposed to 45 °C for 0 min (control), 45 (T1), 60 (T2), and 90 (T3). Then, they recovered under standard conditions (23 °C) for 7 days and the plant fresh weight and the chlorophyll (Chl) content were determined. The Chl content in the disc leaves was measured spectrophotometrically, which was carried out after extraction of the leaf discs with 95% ethanol.

### 4.6. Seed Germination under ABA Treatment

Seeds from Arabidopsis WT and *SlCML39-*overexpressing line OE4-8 and OE7-9 were sown on 1/2 MS medium in the same plates with 0, 1.0, or 1.5 µM ABA. Seeds were incubated in the dark at 4 °C for 3 days to break dormancy, then moved to a growth chamber at 23 °C under a 16-hour-light/8-hour-dark photoperiod. The germination (radicle emergence) was recorded every day. On the 10th day, photos were taken to show the growth, and the numbers of cotyledon with open and green leaves were also recorded.

### 4.7. ABA Content

Seven-day-old seedlings of *SlCML39*-overexpressing lines OE4-8 and OE7-9 and WT plants were exposed to HT (42 °C) for 2 h. After hormone extraction and solid phase extraction, concentration, and redissolution, ABA was detected qualitatively and quantitatively by UPLC-ESI-MS/MS according to the method of Chen et al. [65] with some modifications. The samples were analyzed using an ACQUITY UPLC H-Class system (Waters, Milford, CT, USA).

### 4.8. RNA-Seq

According to Lu’s experimental design of analyzing HT response by RNA-seq [29], we prepared two separate cDNA libraries: 7-day-old WT plants under HT stress (HT, 2 h at 42 °C); *SlCML39-*overexpressing line OE7-9 under HT stress (HT, 2 h at 42 °C). Each library has 3 replicates, for a total of six samples. The RNA extractions, as well as the quality and quantity measurements, were carried out as previously described by Ding et al. [66], and sequenced on a BGISEQ-500 platform at Huada Genomics Institute [28]. After preprocessing and quality control, the clean reads were separated from the raw data. After assembly, according to Langmead and Salzberg [67], by using Bowtie2 (v2.2.5), clean reads were mapped to unigenes. The transcript levels were calculated and normalized to FPKM (fragments per kilobase per transcript per million mapped reads) using RSEM software (v1.2.12) [68]. The distribution statistics of the FPKM of genes were visualized as boxplots using R software (version 3.5.2). The |log_2_ ratio| ≥ 1 and adjusted *p*-value ≤ 0.05 as the threshold to judge differentially expressed genes (DEGs). GO enrichment analyses were performed using the OmicShare tools (https://www.omicshare.com/tools/, accessed on 10 August 2021).

### 4.9. qRT-PCR

For *SlCML39* expression, total RNA was extracted from Section 2.2 and Section 2.4 above. To verify the accuracy of RNA-seq data, six DEGs were selected. Independent RNA with three replicates of Arabidopsis leaves (WT, OE7-9) under HT was prepared. The TriZol reagent was used for RNA extraction (Takara Bio, Kusatsu shi, Japan). The cDNA Synthesis SuperMix Kit was used to synthesize the first strand of complementary DNA (cDNA) from 1.0 μg of total RNA. SYBR Premix Ex Taq (Takara) was used to run qRT-PCR (ABI 7500 Real-time PCR Systems, Thermo Fisher Scientific, Waltham, MA, USA). Triplicate measurements were performed. Tomato *UBI* and Arabidopsis *ACTIN* were used as an internal control for tomato and Arabidopsis, respectively. The transcript level was normalized as an internal control. Finally, the relative expression level of each gene was calculated according to the 2^−ΔΔCT^ method. Primer sequences are listed in Table S1.

## 5. Conclusions

In conclusion, this study provides a substantial body to support *SlCML9* as a stress-responsive gene and its role in the negative regulation of HT tolerance. Overexpression of *SlCML39* decreased seed germination and seedling growth under HT stress. Our data propose a mechanism whereby the stress/ABA-responsive genes are regulated by HT through *SlCML39*. A simplified working model is shown in Figure 10, which proposes a new pathway combined with the complex ABA and HT response networks in plants. These findings also provide an exciting opportunity for the exploitation of *SlCML39* as a potential modulator for plant HT tolerance as well as abiotic stresses.

## Figures and Tables

**Table 1:** Different expressed genes in 35S: *SlCML39* compared to wild-type *Arabidopsis thaliana* under high temperature (padj < 0.05; |fold change| > 2).

GeneID	Fold Change	padj	Symbol	Description
AT3G09940 **	−1.96	0.000	MDAR3	Monodehydroascorbate reductase 3
AT4G21830 *^,^**	−1.92	0.000	MSRB7	Methionine sulfoxide reductase B7
AT1G02920	−1.55	0.005	GSTF7	Glutathione S-transferase 7
AT1G53903 *	−1.47	0.015		Linoleate 9S-lipoxygenase-4 protein
AT1G53885 *	−1.47	0.015		Linoleate 9S-lipoxygenase-4 protein
AT3G50770 *	−1.39	0.010	CML41	Calmodulin-like 41
AT3G44006 *	−1.37	0.038		unknown protein
AT3G15450 *^,^**	−1.36	0.000		Aluminum-induced protein
AT5G52300 *^,^**	−1.36	0.029	RD29B	Low-temperature 65
AT1G24793 *	−1.35	0.019	AtLpxC1	lipid X C1
AT1G64110 **	−1.34	0.010	DAA1	DUO1-activated ATPase 1
AT2G43820 **	−1.34	0.000	SAGT1	UDP-glucosyltransferase 74F2
AT4G12530 *^,^**	−1.32	0.012	AZI7	Bifunctional inhibitor
AT4G27410 *^,^**	−1.24	0.047	RD26	Responsive to desiccation 26
AT3G44860 *^,^**	−1.22	0.001	FAMT	Farnesoic acid carboxyl-O-methyltransferase
AT5G59220 *^,^**	−1.21	0.000	SAG113	Highly ABA-induced PP2C gene 1/HAI1
AT3G63060 *^,^**	−1.21	0.020	EDL3	EID1-like 3
AT5G02020 *^,^**	−1.19	0.000	SIS	Salt-Induced Serine-rich
AT3G48360 *^,^**	−1.18	0.039	ATBT2	BTB and TAZ domain protein 2
AT1G05100 *^,^**	−1.15	0.021	MAPKKK18	MAP kinase kinase kinase 18
AT1G23390 *^,^**	−1.12	0.000	KFB	A kelch domain-containing F-box protein
AT3G21220	−1.11	0.000	MKK5	MAP kinase kinase 5
AT5G15960 *^,^**	−1.10	0.001	KIN1	Stress-responsive protein (KIN1)
AT5G15500 *^,^**	−1.07	0.000		Ankyrin repeat family protein
AT5G67480 *^,^**	−1.07	0.001	ATBT4	BTB and TAZ domain protein 4
AT3G27250 *^,^**	−1.06	0.009	DIL4	ABA-induced transcription repressor
AT5G22920 *^,^**	−1.01	0.004	AtRZPF34	RING-type Zinc finger protein
AT1G24260 *	−1.00	0.008	SEP3	MADs box transcription factor
AT3G29030 *^,^**	1.01	0.000	ATEXP5	Expansin A5
AT3G56360 *	1.02	0.000		Hypothetical protein
AT1G35260 *	1.04	0.033	MLP165	MLP-like protein 165
AT3G27650 *	1.13	0.024	LBD25	LOB domain-containing protein 25
AT2G43870 *	1.21	0.001		Pectin lyase-like superfamily protein
AT2G41510 *	1.21	0.010	ATCKX1	Cytokinin oxidase
AT2G38530 *	1.24	0.000	ATLTPI-5	Lipid transfer protein 2
AT1G13740 *^,^**	1.26	0.001	AFP2	ABI five binding protein 2
AT1G44800 *^,^**	1.30	0.000	SIAR1	Siliques Are Red 1
AT2G32990 *^,^**	1.30	0.000	GH9B8	Glycosyl hydrolase 9B8

Fold change indicates values from the three independent replicates of up/down-regulation in 35S: *SlCML39* line OE7-9 compared with wild-type (Col-0) under high temperature. *^,^** Indicates that the gene expression was induced by high temperature and ABA, respectively, based on the GO biological process (response to ABA) or the studies of high temperature-induced transcriptomics in Arabidopsis (Tables S3 and S4). All 38 gene expression data are in Table S5.

## Data Availability

The data presented in this study are available in the article and Supplementary Materials.

## Supplementary Materials

The following are available online at https://www.mdpi.com/article/10.3390/ijms222111479/s1.
