# Supplementary material for: Characteristics of SlCML39, a Tomato Calmodulin-like Gene, and Its Negative Role in High Temperature Tolerance of Arabidopsis thaliana during Germination and Seedling Growth
Source: Int J Mol Sci. 2021 Oct 25;22(21):11479. doi: 10.3390/ijms222111479 (PMC8584099; doi:10.3390/ijms222111479)
Supplement: Supplementary file 1 [file ijms-22-11479-s001.zip › ijms-1416167-supplementary Figures.pdf]

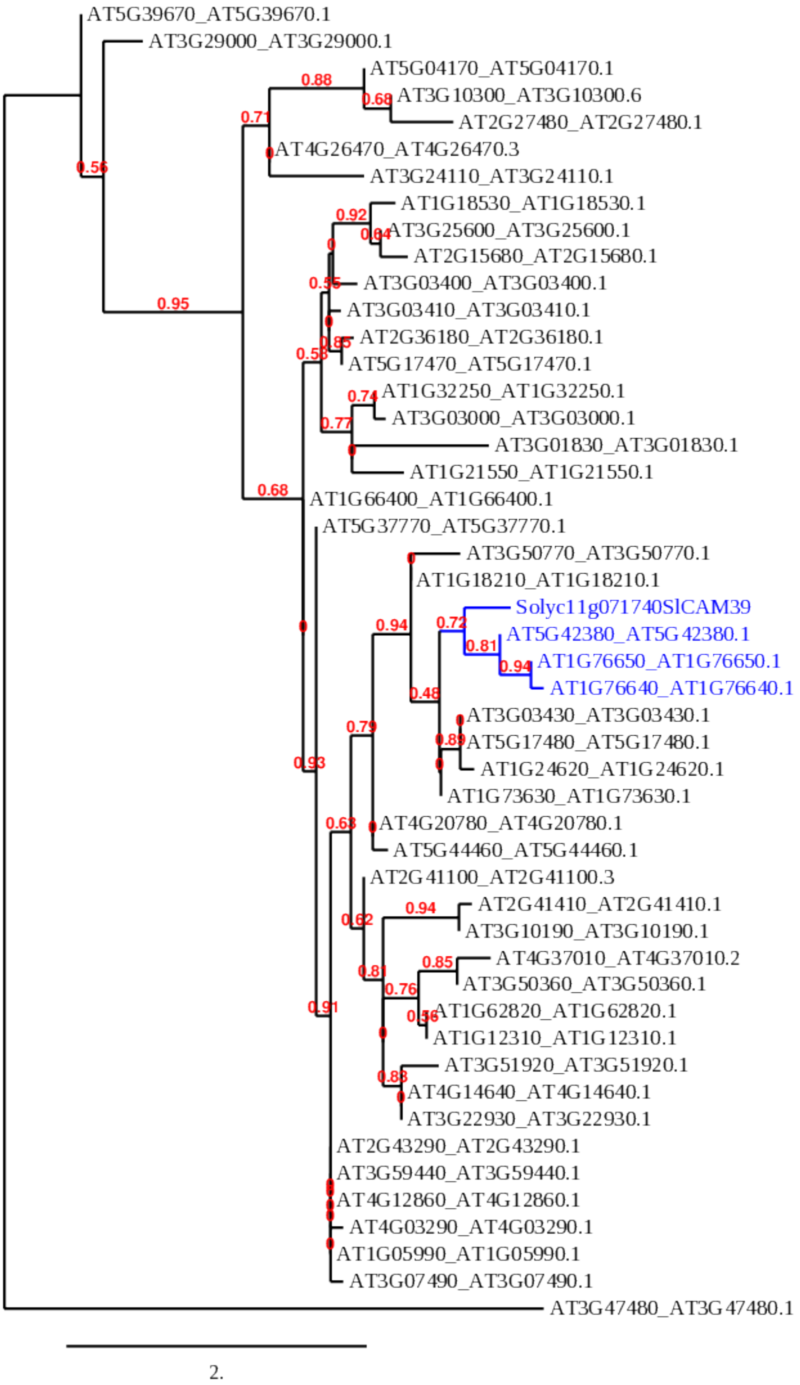

Figure S1. Phylogenetic tree of SlCML39 and its related CML proteins from Arabidopsis. The phylogenetic tree was constructed using MUSCLE/PhyML programs. All protein sequences were download from the Phytozome 13 (https://phytozome-next.jgi.doe.gov/). The blue marker is Arabidopsis CMLs in the same subfamily as SlCML39..

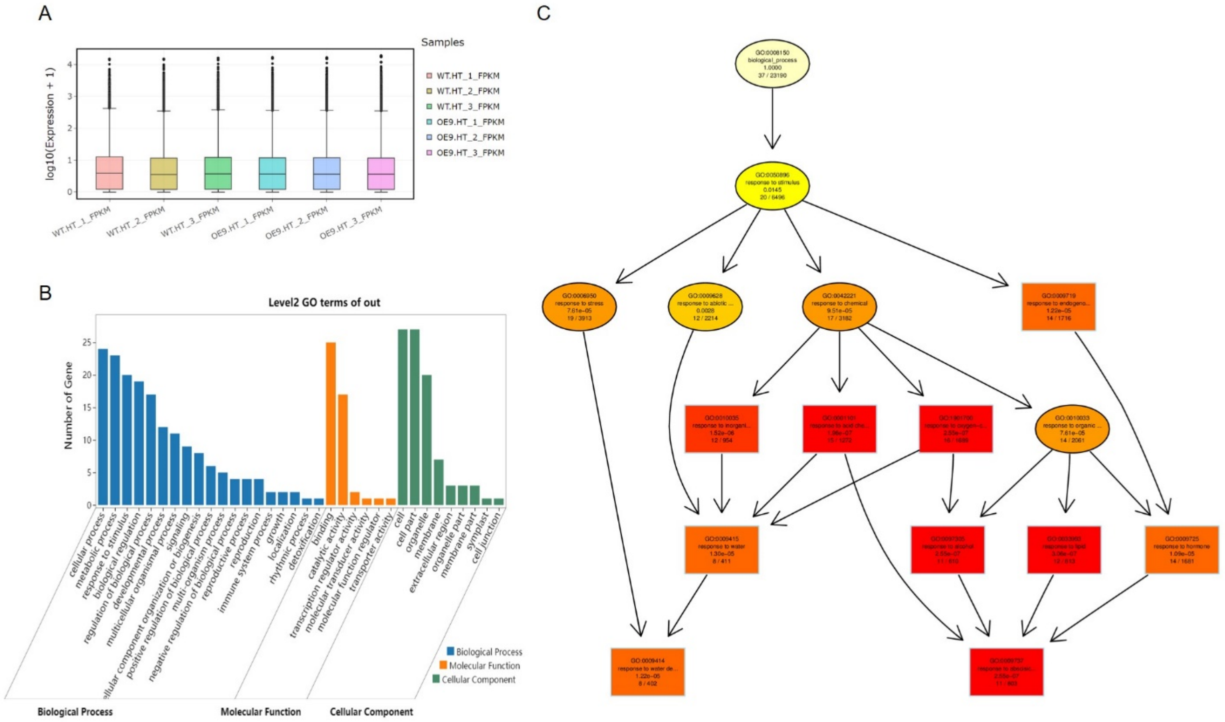

Figure S2. The boxplot and GO analysis of differentially expressed genes (DEGs). A. The boxplot showed the global gene expression level (log10 FPKM) of each sample. The boxplot for each region corresponds to the maximum, upper quartile, median, lower quartile, and minimum FPKM from top to bottom. B. GO enrichment analysis on DEGs. Blue refers to terms relating to biological processes, yellow refers to terms relating to cellular components, and green refers to terms relating to molecular function. C. GO enrichment.

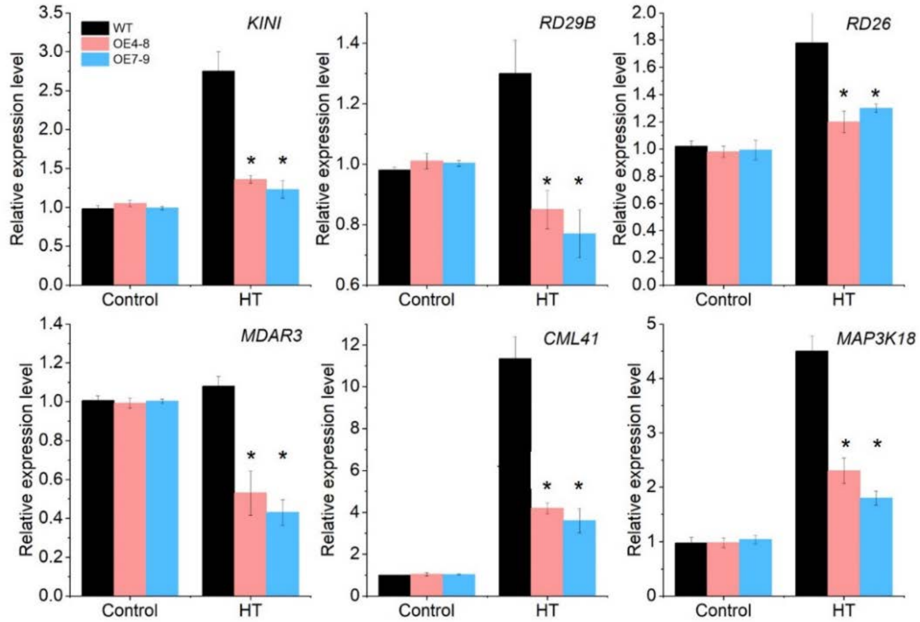

Figure S3. The transcript levels of selected stress responsive genes under high temperature (HT). qRT-PCR analyses of *KIN1*, *RD29B*, *RD26*, *MDAR3*, *CML41*, and *MAP3K18* were carried out in one-week-old wild type and two *SlCML39* overexpressing lines (OE4-8, OE7-9) were treated at 42 °C for 2 h. All rosette leaves were collected for RNA extraction. *ACTIN* gene is used as an internal control. The bar represents the mean ± SD values of three replicates (n=3). *P < 0.05 by Student's t-test.

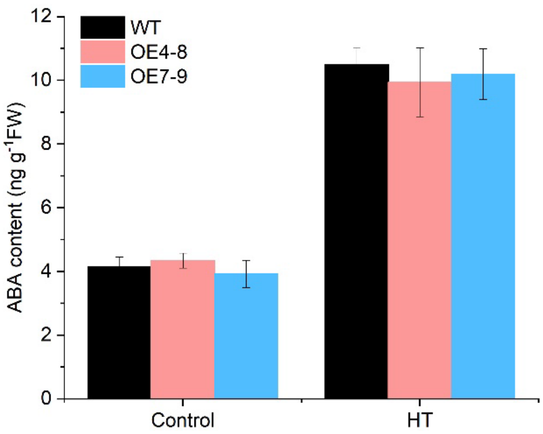

Figure S4. The content of abscisic acid (ABA) in *Arabidopsis* leaves exposed to high temperature (HT). 7-day-old seedlings of*SlCML39*-overexpressing lines (OE4-8 and OE7-9) and wild type (WT) plants were exposed to HT (42 °C) for 2 h and sampled for ABA analysis. The bar represents the mean ± SD values of three replicates (n=3). *P < 0.05 by Student's t-test.
